# Dual X-Ray and Optical System for In Situ Monitoring of Solids Fraction in Tailings

**DOI:** 10.3390/s26175645

**Published:** 2026-09-05

**Authors:** Talwinder Kaur Sraw, Bo Yu, Xiaoxuan Liu, Jason Ng, Andrea Sedgwick, Manisha Gupta, Robert Fedosejevs, Ying Yin Tsui

**Affiliations:** 1 Department of Electrical and Computer Engineering, University of Alberta, 9211 116 Street NW, Edmonton, AB T6G 1H9, Canada; 2 Centre for Energy and Environmental Sustainability, Northern Alberta Institute of Technology, 10210 Princess Elizabeth Ave NW, Edmonton, AB T5G 0Y2, Canada

**Keywords:** radioactive sensing elements, infrared laser-based detection, X-ray attenuation monitoring, multi-pixel photon counters, scintillating crystalline materials

## Abstract

**Highlights:**

**What are the main findings?**

- A full operational system was successfully demonstrated for in situ measurements of solids content in a large pilot-scale settling column.
- In situ hybrid optical and gamma-ray techniques provide accurate solids content measurements that agree well with conventional laboratory gravimetric measurements.

**What are the implications of the main findings?**

- The technology enables rapid measurement of solids’ depth profiles in settling ponds without the need for time-consuming and costly sampling followed by subsequent laboratory analysis.
- The technology can facilitate better monitoring and management of oil sands tailings, resulting in improved process efficiency and improved environmental performance.

**Abstract:**

A sensing apparatus was developed to quantify solids fraction within a 3 m test column, demonstrating real-time monitoring of tailings sedimentation. This integrated system employs a low-power infrared laser coupled with scattering detectors alongside a low-activity radioactive source and detector. Both methodologies utilize non-destructive principles to evaluate the concentration of solids. Specifically, X-ray transmission through the medium provides a measure of the solids fraction, as X-ray attenuation corresponds to the solids percentage. Concurrently, the optical component detects radiation backscatter from the sample. To ensure precision, both instruments underwent calibration using reference materials. Experimental evaluations were conducted using two configurations: a fixed array and a depth-profiling setup. The former utilized a 2.5 m plastic column with integrated sensors placed within a settling vessel to acquire data at discrete depths. Conversely, the depth-profiling configuration involved a submersible mobile unit lowered into the column to characterize the solids profile at arbitrary depths.

## 1. Introduction

The oil industry in Alberta is one of the largest oil-producing sectors in the world. In the Wood Buffalo region, bitumen is extracted from mined oil sands ore and processed in mineral extraction facilities. The ore is combined with hot water to separate the bitumen from the sand, silt, and clay fractions. A small amount of chemicals is added to facilitate separation. This process generates a tailings slurry comprising water, sand, fine solids, and residual bitumen. The slurry is deposited in tailings ponds to facilitate settlement. The sand settles quickly, while the fine solids take considerable time to consolidate. This process uses large volumes of water [1], which must be recycled and reused to minimize environmental impacts. Proper tailings management strategies are essential to maximize water reuse and facilitate reclamation [2,3].

The fine tailings remaining in the ponds after water recovery, commonly known as Mature Fine Tailings (MFTs), can take decades to settle. Consolidation of MFTs is typically a non-uniform phenomenon; different solid concentration layers can be found along the depth of the pond [4,5]. Several tailings treatment methods are currently being investigated and/or implemented by the industry to facilitate consolidation, such as centrifuge dewatering, polymer flocculation, and microbial acceleration [6,7,8,9]. Understanding innate MFT behavior and assessing accelerated consolidation techniques both require testing field slurry samples for various parameters, such as particle size, soil composition, and solids concentration [10]. The solids concentration, in particular, is a key parameter for monitoring and managing tailings storage, settling, treatment, dredging, pumpability, and pipeline transport. The gravimetric method is still the principal method used to survey the pond. Directive 85 (AER, 2022) requires companies to provide the “composition of each legacy tailings deposit and pond.” This results in extensive pond surveys requiring the extraction of samples and sending them to labs for analysis [11]. This process takes a lot of time, personnel, and expenses. A real-time in situ device that is relatively low cost, decreases personnel, and provides real-time data would be very desirable for field operations.

A variety of diagnostic tools [12,13,14,15,16,17,18,19,20,21,22,23,24] have been proposed and investigated to provide real-time in situ monitoring of industrial slurries. These can provide critical process data, but each of these techniques faces constraints that limit their deployment in the field. Ultrasonic probing is a promising technique, but high-density slurries rapidly attenuate the acoustic waves, limiting measurements at depths where solids concentrations may be high. Additionally, temperature shifts and varying particle sizes alter wave propagation, requiring correction algorithms, while bulky hardware makes miniaturization difficult [12,13,14,15]. Microwave technologies [16,17] are also sensitive to water conductivity and particle distribution size; thus, they may have limitations on accurate measurements of the solids content of industrial slurries because these two properties can vary considerably. Electrical resistive tomography [18,19,20] and conductive technologies depend on the salinity and temperature of the sample, which can be quite variable in field samples. To date, optical techniques have focused on hyperspectral imaging. This technique requires large initial data sets to train site-specific neural networks [21,22,23] and is sensitive to fouling of windows from contamination, which can easily mask or modify spectral features. Measuring thermal conductivity via pulsed heating [24] is another technique, which requires measuring temperature changes with sub-degree accuracy and may have limitations from relatively large environmental temperature fluctuations in the field. None of these techniques have seen large-scale deployment in the field as real-time sensors to date. Time domain reflectometry (TDR) and thermal conductivity sensors are also applied in the field to measure bulk water content in tailings soil [25,26]. These methods face similar challenges when applied to fine oil sands tailings, where trapped pore water and high salinity can disturb their effectiveness [27]. Our sensor techniques based on NIR backscattering [11,28] and low-intensity gamma-ray transmission [29] are insensitive to variations in the slurry particle size distributions, temperature, salinity, and chemical composition. They can be implemented using relatively simple and low-cost electronic systems and do not require complex model-based analysis to extract the results. The sensor systems can be further miniaturized to a scale size of a few inches.

This paper aims to develop a technology with a hybrid optical and gamma-ray system for subsurface solids content analysis in the settling pond. This technology is based on optical [11,28] and safe gamma-ray [29,30,31] methods. It enables in situ, real-time measurements of solids content at various depths within a tailings pond. An instrument utilizing this technology can be adopted for multiple applications, including use in oil sands tailings and other mine tailings [32]. The implementation of this in situ solids content measurement technology would allow oil sands operators to make real-time adjustments to the design of their tailings management projects, resulting in more efficient dewatering and reclamation activities and leading to enhanced environmental performance. Furthermore, this integrated sensing architecture can be refined into a reduced physical footprint, facilitating streamlined field deployment and operational integration.

**Figure 1:**
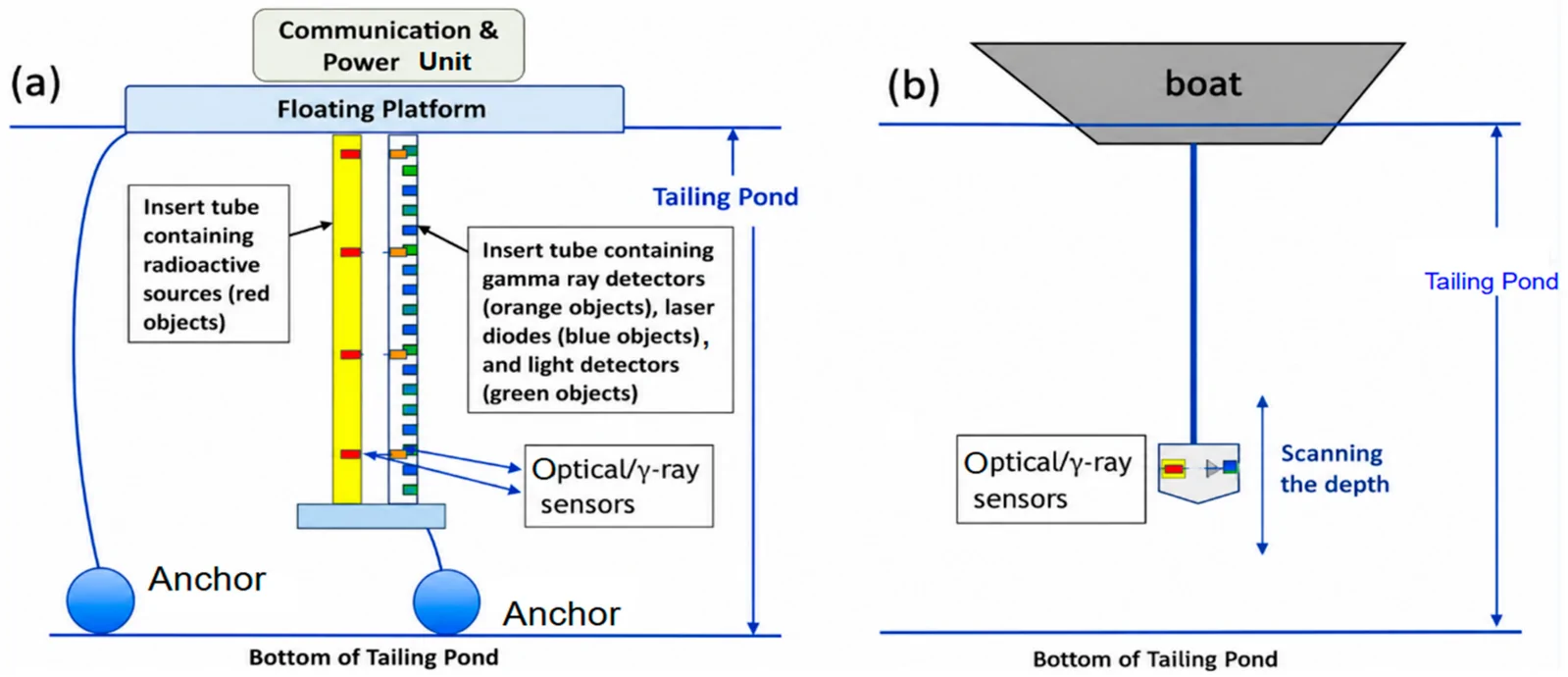
Conceptual field systems: (**a**) stationary solids content analyzer (ST-SCA) and (**b**) depth-scanning solids content analyzer (DS-SCA).

Two concepts for field implementation have been developed. The first concept, called the stationary solids content analyzer (ST-SCA), consists of multiple optical and gamma-ray sensors placed at various heights within a long, watertight insertion tube. This insertion tube can be installed at a fixed floating location in a tailings pond to continuously monitor the solids content at different depths. The stationary system is beneficial due to its continuous operation. All the data processed by the microprocessor for both techniques is saved on an electronic device/laptop. Data can also be transferred to a remote location. Once the setup is installed inside the pond, there is no need for personnel to take samples unless a technical issue occurs. Multiple units may be required to monitor different locations to cover the entire tailings pond. The second concept, called the depth-scanning solids content analyzer (DS-SCA), comprises an optical sensor and a gamma-ray sensor housed in watertight housing. This unit can be mounted on a boat and lowered to the desired position in a tailings pond to measure solids content at various depths. The DS-SCA is capable of scanning different locations as needed and is designed to be deployable for use anywhere in the tailings pond. This is particularly useful for dredging operations personnel who may rely on limited and outdated information. Tailings management can become more efficient, as the unit can aid in targeting a specific solids content range, which can significantly maximize efficiency in chemical dosing, thickening, dewatering, and other processes. Schematic diagrams illustrating these two concepts are shown in Figure 1.

Prototypes for these two concepts were built and tested in a 3-m-high pilot-scale settling column containing tailings. Settling behavior is a slow process, and fine tailings take weeks to months to settle. Each detector prototype experiment was carried out over a period of a few months to study the settling dynamics.

## 2. Experimental Setup

**Figure 2:**
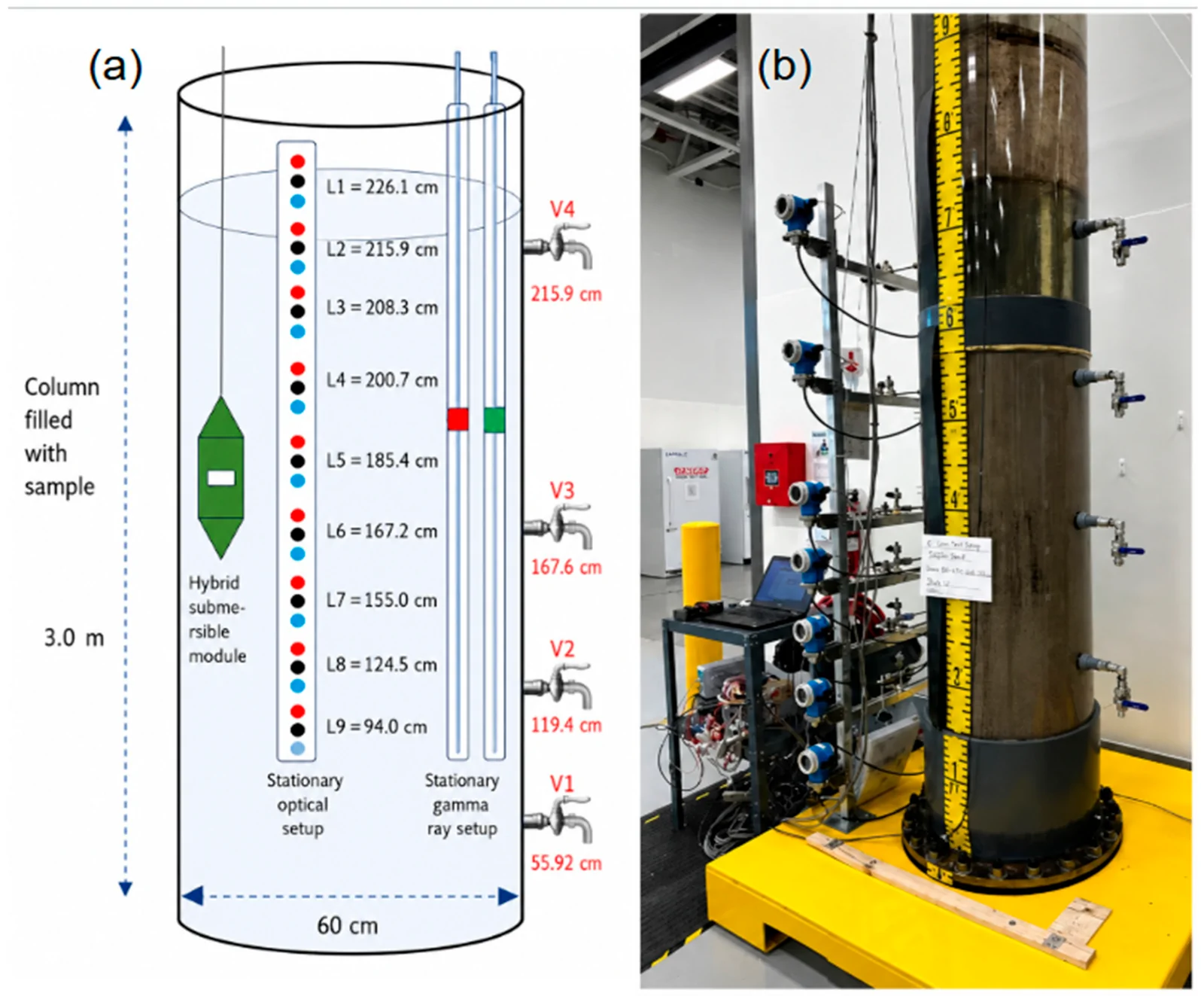
(**a**) Schematic diagram and (**b**) experimental setup for testing the two SCA prototypes.

**Figure 3:**
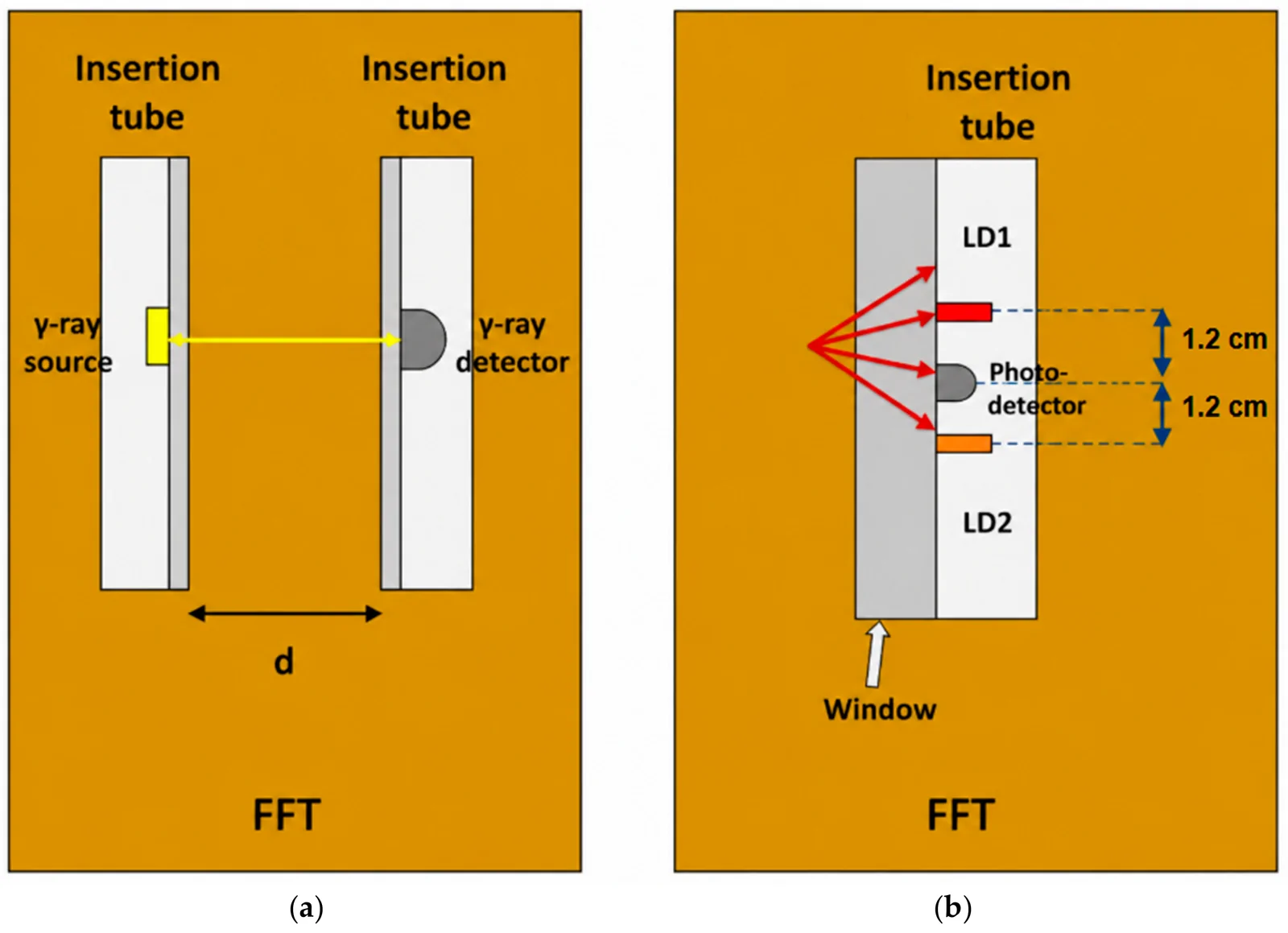
Schematic diagram of sensors in ST-SCA: (**a**) γ-ray sensor with a low-activity source and a detector and (**b**) optical sensor comprising LD1 and LD2 laser diodes with wavelengths of 1550 and 980 nm, respectively, and a photodetector (PD).

The column setup was designed to study the long-term settling and consolidation behavior of tailings and simulate the conditions when the analyzer is submerged in the tailings ponds (Figure 1). The prototypes were developed to monitor settling behavior and estimate solids content at different depths. Details of the settling tank setup for testing the two prototypes, ST-SCA and DS-SCA, are shown in Figure 2. The ST-SCA (Figure 2a) prototype consists of nine optical sensors and a movable gamma-ray sensor. The gamma-ray sensor and source position can be fixed to predetermined positions. The optical sensors are placed inside a long, watertight plexiglass tube. Each optical sensor comprises two laser diodes and a photodetector to measure backscattered light from the tailings. Figure 3b illustrates the principles of the optical sensor. The sampling time for the optical sensor was 20 s. Only one laser diode was activated at a time to prevent signal mixing from the two wavelengths. The locations of the photodiodes in the optical sensors are labeled L1 to L9 in Figure 2a. The distances from the bottom of the column to L1 through L9 are 226.1, 215.9, 208.3, 200.7, 185.4, 167.2, 155, 124.5, and 94 cm, respectively. The gamma-ray sensor, which can be moved up and down, was placed inside a pair of long, watertight plexiglass tubes. The gamma-ray sensor consisted of a low-activity 1 μCi Ba-133 source (more than ten times below the licensing limit) and a custom-built, low-cost, compact gamma-ray detector [31]. The diagram in Figure 3a illustrates the principles of the gamma-ray sensor. The sampling time for the gamma-ray sensor was 15 min to obtain a data point with a good signal-to-noise ratio (SNR). Four valves on the column were used to extract settling FFT samples for gravimetric comparison with the sensor data. The valve locations are labeled V1 to V4 in Figure 2. The distances from the bottom of the column to V1 through V4 were 55.9, 119.4, 167.6, and 215.9 cm, respectively. For gravimetric results, a CEM SMART System 5 moisture analyzer (CEM Corporation, Matthews, NC, USA) was used to measure the solids content. Approximately 3 to 5 g of FFT was used. The moisture analyzer uses dual-frequency microwaves as the energy source to volatilize polar compounds, including water. Because the remaining solids do not absorb microwave energy and remain cool, the solids content can be determined from the measured weight loss. It is important to note that the solids content determined from this method includes the mineral solids and bitumen. Based on the Dean–Stark extraction method, which is an industry standard, the bitumen content of the FFT used in this study was 1.05 wt%.

The setup consisted of a 3 m tall column with a 0.6 m diameter. A DS-SCA (hybrid submersible module) and ST-SCA (stationary optical setup and stationary gamma-ray setup) were placed inside the column, as shown in Figure 2. The locations of the optical sensors (red circles represent 1550 nm LD, blue circles represent 980 nm LD, and black circles represent PD) are labeled as L1 to L9, with the distances measured from the bottom of the column. The γ-ray sensor, represented by the red object, consists of the Ba-133 γ-ray source, and the green object represents the γ-ray detector, which can be moved together up and down and is located inside a pair of watertight tubes. The 4 valves on the right-hand side outside of the column at various heights from the bottom of the column were used to extract the FFT samples at various depths for comparison with the sensor data.

**Figure 4:**
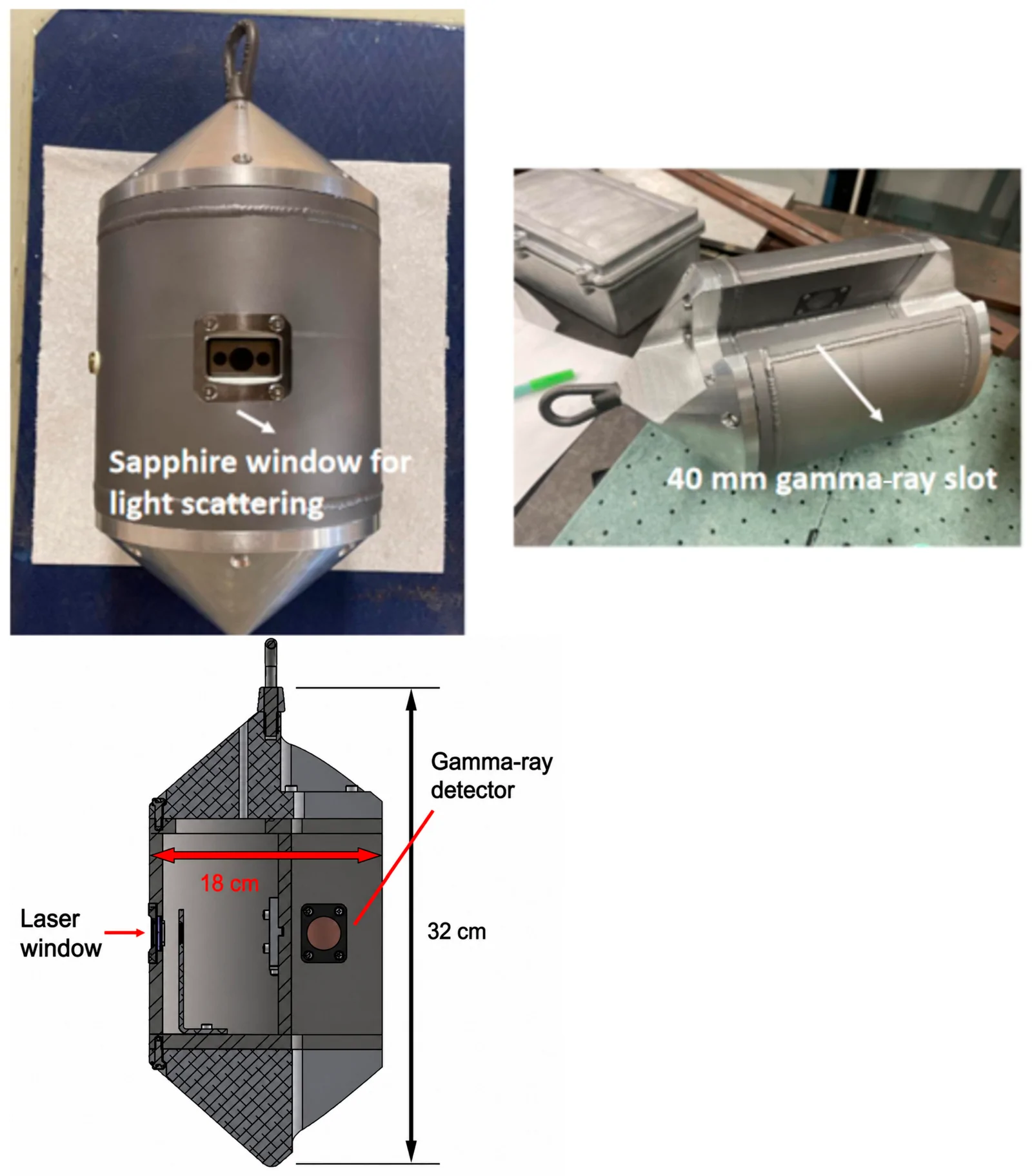
Pictures of the DS-SCA. The main body of the cylindrical housing is 18 cm in diameter and 32 cm in length.

**Figure 5:**
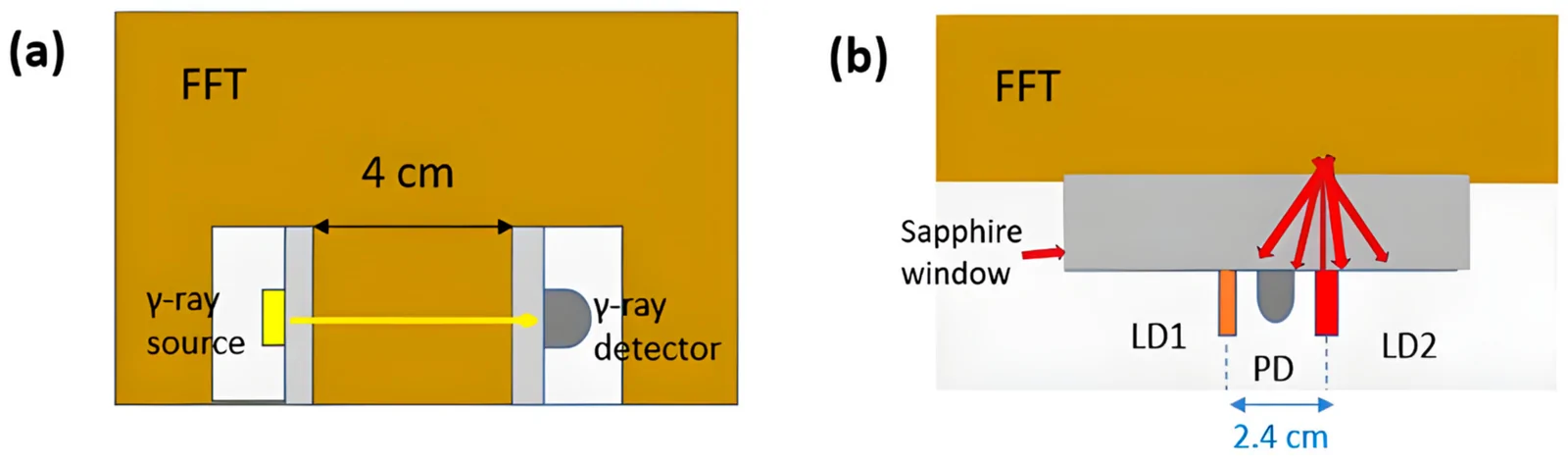
Schematic diagram of sensors in DS-SCA: (**a**) γ-ray sensor with a low-activity source and detector and (**b**) optical sensor comprising LD1 and LD2 laser diodes with wavelengths of 1550 and 980 nm, respectively, and a single photodetector (PD).

A picture of the DS-SCA prototype is shown in Figure 4. The main components of the DS-SCA include two laser diodes with a photodetector to measure backscattered light from the tailings and a gamma-ray sensor consisting of a safe low-strength 1 μCi Ba-133 source. The submersible module was a custom-made, watertight unit that is specially designed for tailings operations. Figure 5 illustrates the two sensors in the DS-SCA. The sampling time and operation were similar to that for the stationary system (ST-SCA). The submersible module is portable for use at different locations.

### 2.1. Calibration of the Sensor System

FFT and kaolin samples were used as test and calibration samples, respectively. Commercially available kaolin samples of different particle sizes (200, 600, and 2500 nm) were purchased from BASF (McIntyre, GA 31054, USA) for the experiment. A 17.5 wt% FFT sample from Kearl mine was provided by an oil sands operator. The particle size of the FFT sample ranged from 1 to 100 µm. The optical sensors measured the scattered light signal, which was amplified and read out in volts. The gamma-ray sensor measured the transmitted X-ray pulse signals, which were amplified and read out in volts for the peak pulse heights corresponding to X-ray photon energy. Both measurements were converted into solids content expressed as a weight percentage (wt%). A calibration curve was obtained from lab tests with samples of different weight percentages. X-ray signals with pulse heights corresponding to 30 keV were counted per unit time and compared with a starting count rate in pure water.

#### 2.1.1. Optical Sensor

**Figure 6:**
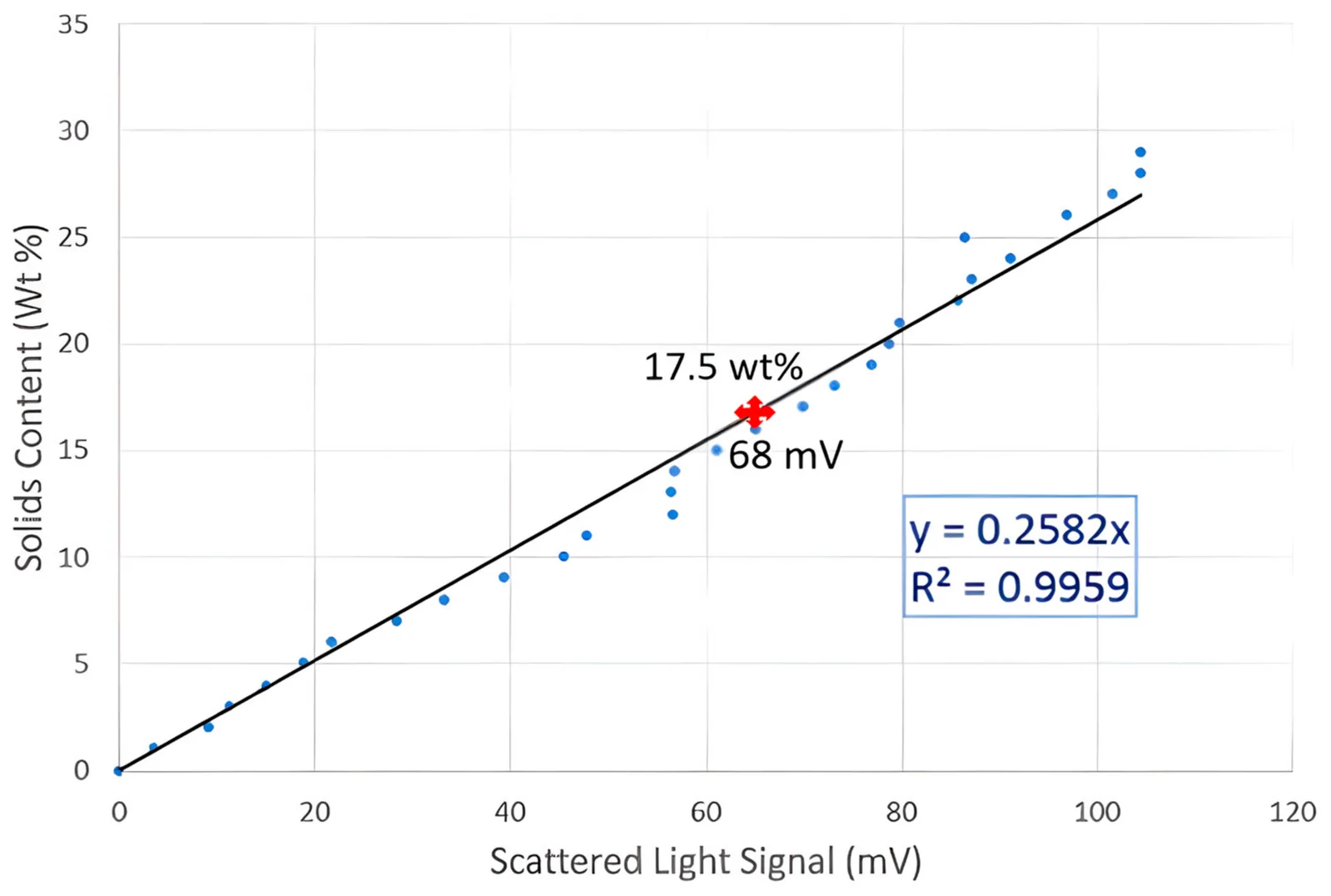
A plot of the FFT solids content as a function of the 1550 nm wavelength laser light scattering signal. The blue circles represent experimental data. The black solid line is the linear regression fit. The equation of the fit and the square of the correlation coefficient (R^2^) are given in the plot.

The scattered light intensity signal at a wavelength of 1550 nm was obtained using a previously reported “ring setup” [33] as a function of FFT solids content and is shown in Figure 6. The detector was positioned 20 degrees from the laser axis and 12 cm from the sample surface. Various wt% FFT samples were prepared by diluting a 30.4 wt% FFT sample taken from the bottom level of the 3-m settling column after the initial sample of the tailings had settled. At the beginning of the experimental run, the 17.5 wt% FFT sample was first homogenized and then poured into the settling column. The initial light scattering signal for each optical sensor provided a 17.5 wt% scattered light signal value. The 17.5 wt% light scattering value for the “ring setup” was 68 mV.

#### 2.1.2. Gamma-Ray Sensor

**Figure 7:**
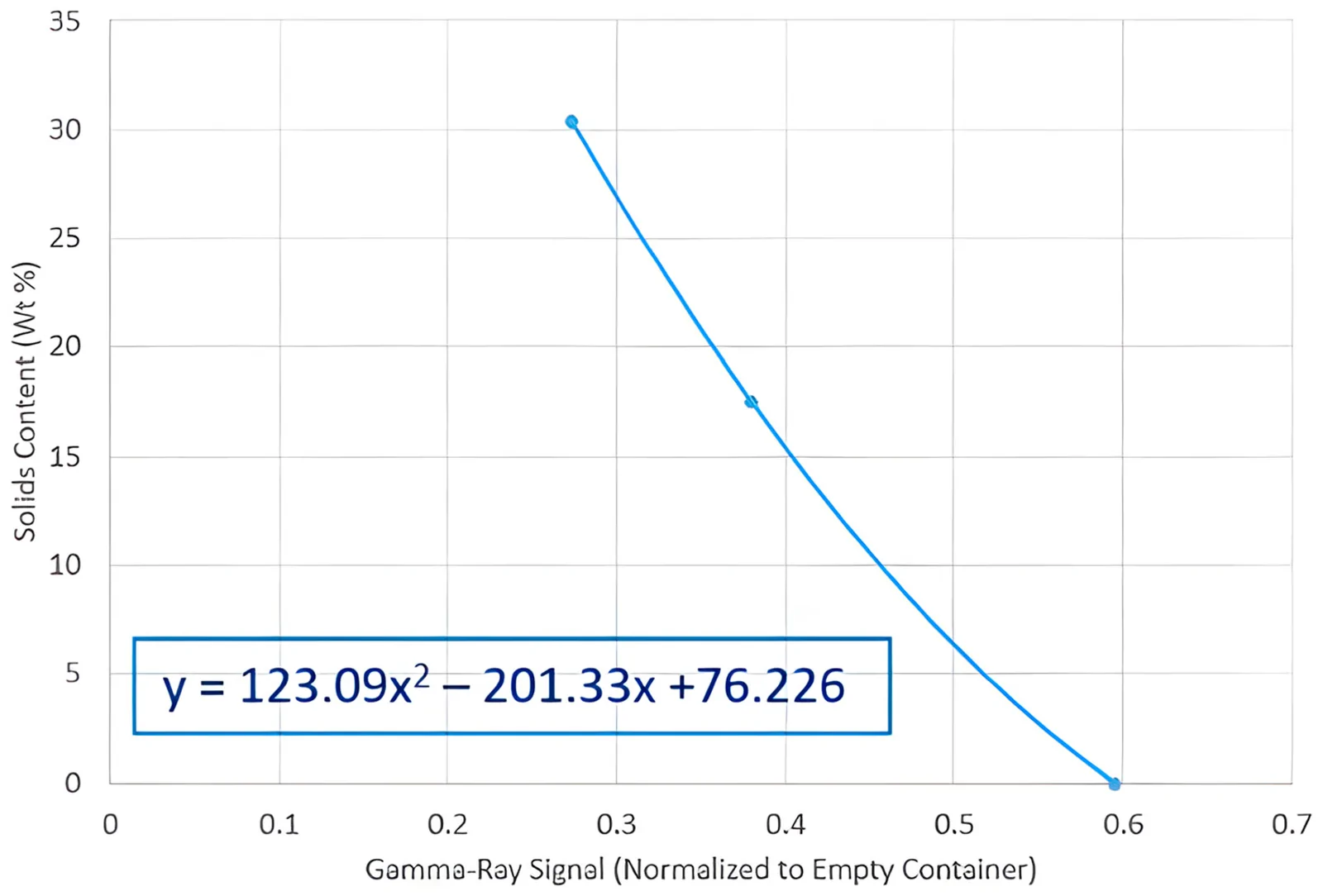
A plot of the FFT solids content as a function of γ-ray transmission at 30 keV. The blue circles represent experimental data. The blue solid curve is the quadratic fit. The equation of the fit is displayed in a box in the lower left-hand corner of the plot with a blue boundary.

The signals for the ST-SCA γ-ray sensor at FFT solids contents of 0 wt% (i.e., released water), 17.5 wt% (value when FFT was initially poured into the column), and 30.4 wt% are plotted in Figure 7. The latter solids content value was obtained from an FFT sample extracted by V1 after a long period of settling using the gravimetric method. V1 is the value closest to the column bottom. A best-fit quadratic equation was used in the calculation to convert the transmission signal of the γ-ray sensor to FFT solids content. In our previous study [29] using a setup similar to the current study, measurements and Monte Carlo GEANT4 simulations indicated that the variation in the 30 KeV γ-ray signals as a function of FFT solids content was non-linear and best-fitted using a quadratic dependency. Each data point in the γ-ray calibration curve was represented by the average of 5 measurements with a small standard deviation of less than 3 wt%.

## 3. Results

### 3.1. Mudline Detection

**Figure 8:**
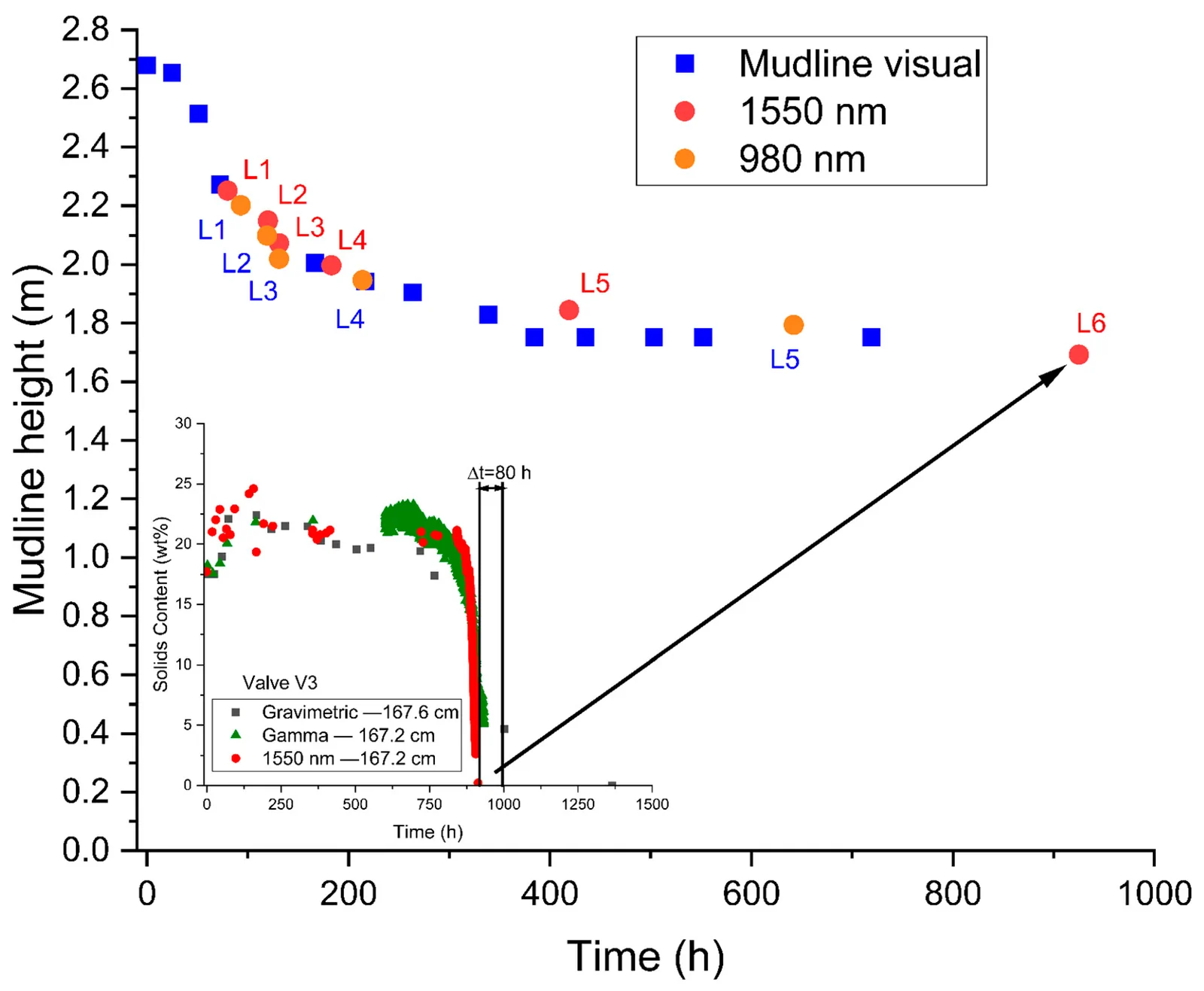
Mudline detection by visual inspection (blue squares) and ST-SCA data at various depths (circles). The temporal evolution of the solids content in wt% for the L6 1550 nm optical sensor is shown in the insert plot. The blue dashed line indicates the time when the mudline crosses this level.

The prototypes of the ST-SCA and DS-SCA concepts (Figure 1) were built and tested in a 3-m-tall pilot-scale settling column at NAIT (Figure 2). The mudline separates the water layer and the settled solids layer. An intermittent turbid layer may be present between the mudline and clear water. Generally, the intermittent layer has a solids content of less than 5 wt% and consists of mostly settling fine solids. In oil sands operations, the mudline depth is required for dredging to extract the released water above the mudline for recycling and reuse in extraction. The location of the mudline in the column as a function of time was determined from the data collected by ST-SCA’s optical sensors and confirmed by visual inspection. The mudline position relative to the bottom of the column was measured regularly (shown as blue squares in Figure 8). The times at which the mudline crossed each ST-SCA optical sensor (1550 nm indicated by the red circle and 980 nm indicated by the orange circle in Figure 8) were also determined when the solids content values dropped to zero (see inset diagram in Figure 8). The results show good agreement between the optical sensor measurement of the mudline and the actual mudline determined by visual inspection. The distance between the laser and photodiode can introduce some discrepancies in the data used for mudline detection. Comparison of the visually measured mudline and the mudline recorded by the laser data indicates that the optical system achieved an accuracy of 2 cm. The scale used for measuring the height of the mudline can also introduce some errors. All levels (labeled L1, L2, L3, L4, and L5) are aligned with the visual mudline results. The 980 nm wavelength provided very accurate data in the range of 0 to 5 wt% and then became saturated above 8 wt%. Therefore, the 980 nm wavelength laser can be used as a primary detection system for mudline detection and dredging operations, whereas a 1550 nm wavelength is more suitable for solids content measurements. During the experiment, the 980 nm signal increased sharply from 0 to 1 wt%, whereas the 1550 nm signal slowly increased with an increase in solids content. The 980 nm wavelength is particularly effective near the mudline, where a transition layer may exist between the water layer and the clear solids content layer. Because this layer has a very low weight percentage, the 980 nm photodiode can detect it.

### 3.2. Temporal Profile of Solids Content with Stationary Setup

In the tailings pond, the solids content increases with depth. Gravimetric methods are traditionally used to measure the solids content. In this study, the results from the SCA were compared with gravimetric measurements of samples taken at the four outlet valves of the 3 m column.

#### 3.2.1. V1 Valve Position Comparison

**Figure 9:**
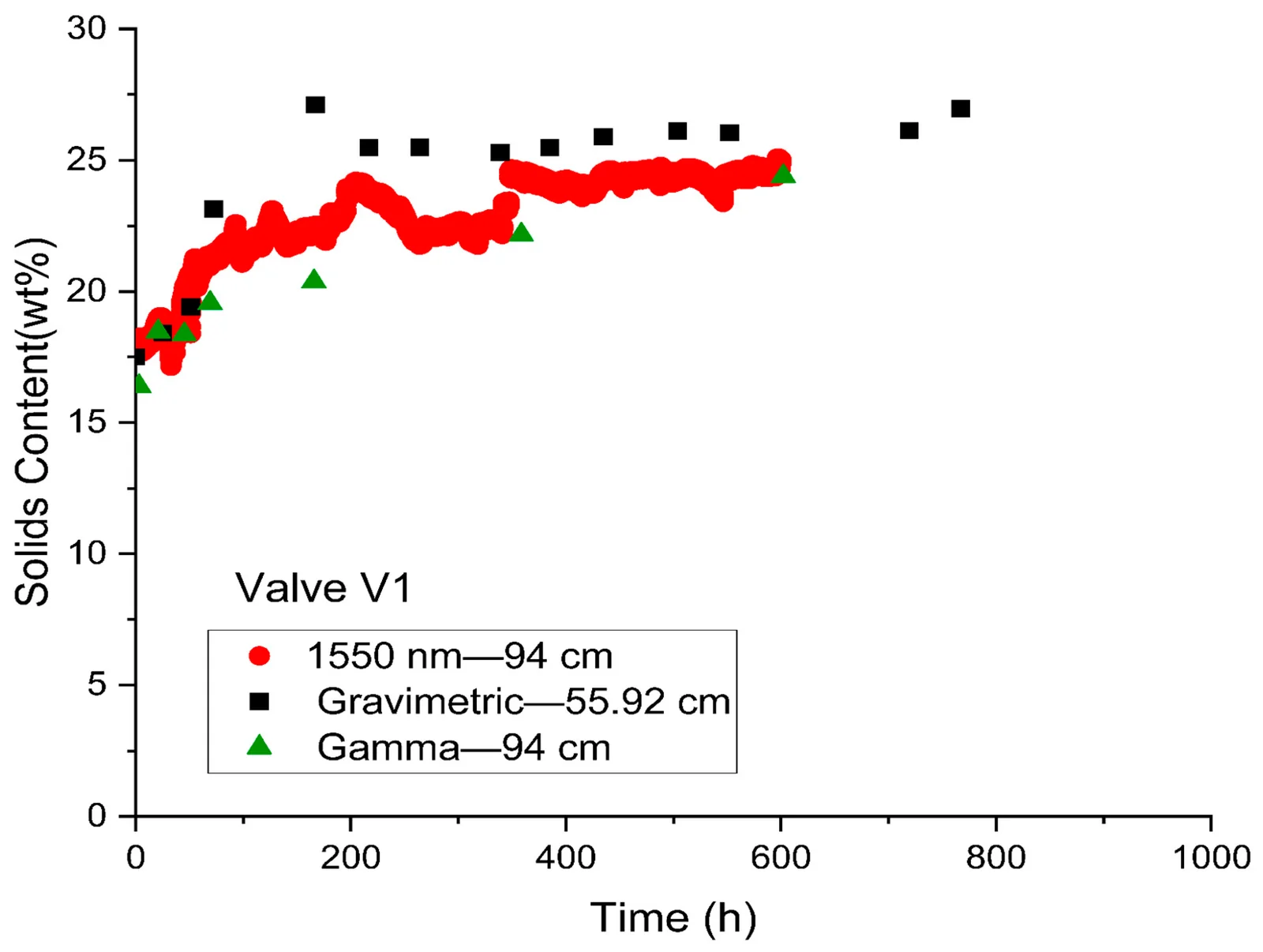
Temporal profiles of solids content measured by the 1550 nm optical (red circle) and γ-ray (green triangle) sensors of the ST-SCA and the gravimetric method on extracted samples (black square) near the height of valve V1.

The temporal profiles of solids content at various depths were measured using in situ optical and gamma-ray sensors and using the gravimetric method on extracted samples. As shown in Figure 9, the temporal profiles of solids content near V1 (bottom valve) were measured by the 1550 nm optical sensor and the gamma-ray sensor of the ST-SCA and compared with those measured using the gravimetric method on extracted samples. The optical and gamma-ray sensors were located 94 cm from the column bottom. The FFT samples used for the gravimetric method were extracted using V1, which was located 55.9 cm from the bottom of the column. The slightly higher values obtained by the gravimetric method may be attributed to the sample being extracted from a location closer to the bottom than the actual positions of the two sensors, which are located approximately 40 cm above the sample extraction position. Once the large particles had settled, there were little fluctuations in the solids content data with longer settling time. The heavy particles settled first, and there were few fluctuations in the data after 200 h. Due to multiple unknown factors and missing corresponding level data, the plot illustrates the data relative to the nearest valve and interpolates the results up to 94 cm.

#### 3.2.2. V2 Valve Position Comparison

**Figure 10:**
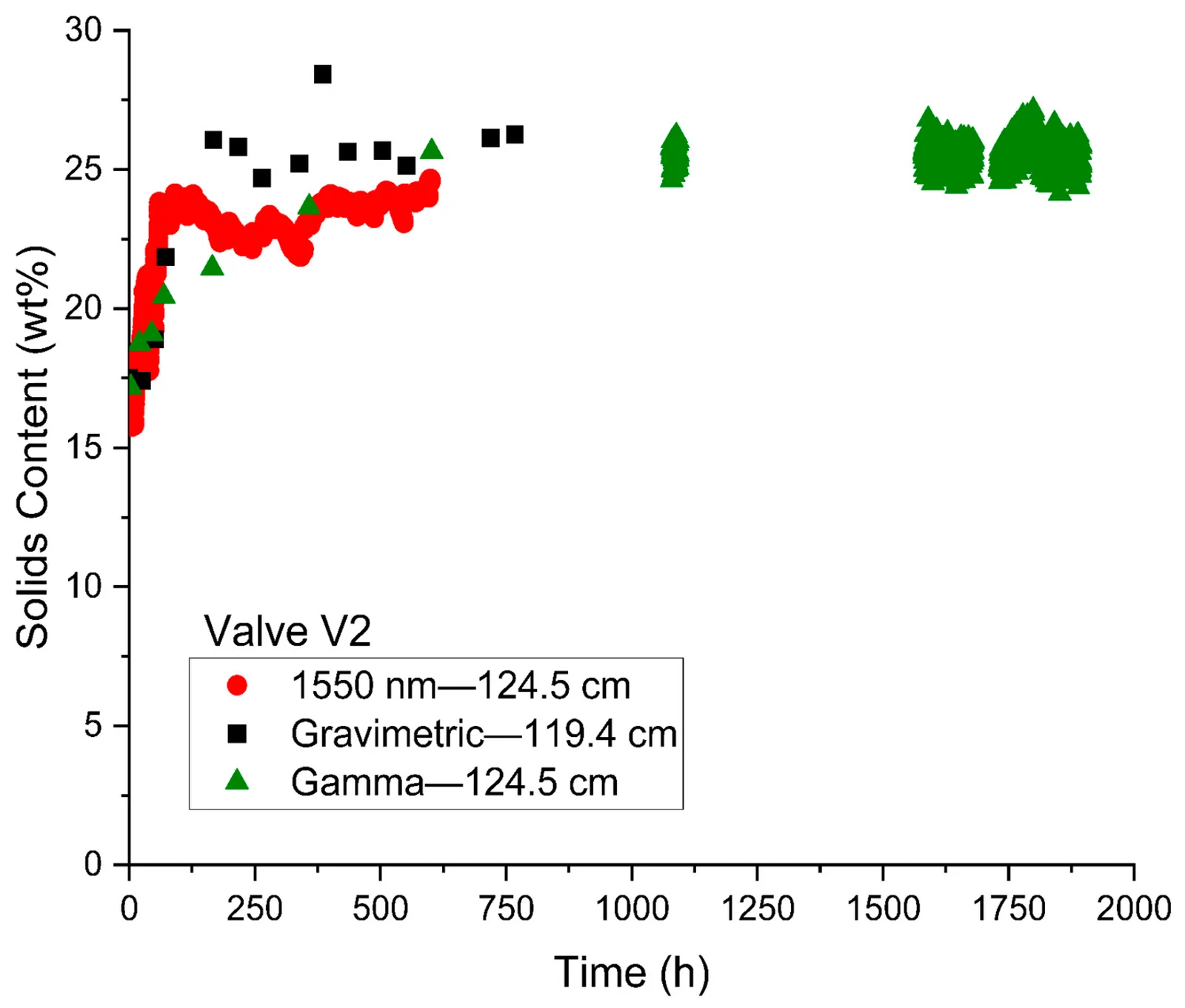
Temporal profiles of solids content measured by the 1550 nm optical (red circle) and γ-ray (green triangles) sensors of the ST-SCA and the gravimetric method on extracted samples (black square) near the height of outlet valve V2.

As shown in Figure 10, the temporal evolution of the solids content near V2 was measured by the 1550 nm optical sensor and the gamma-ray sensor of the ST-SCA and compared with those obtained by the gravimetric method on extracted samples. The optical and gamma-ray sensors were located 124.5 cm from the bottom of the column. The FFT samples used for the gravimetric method were extracted at V2, which was located 119.4 cm from the column bottom. The gravimetric method obtained higher wt% values than those from SCA. Again, this is likely because the gravimetric samples were extracted at a position 5 cm lower than the optical and gamma-ray sensors.

#### 3.2.3. V3 Valve Position Comparison

**Figure 11:**
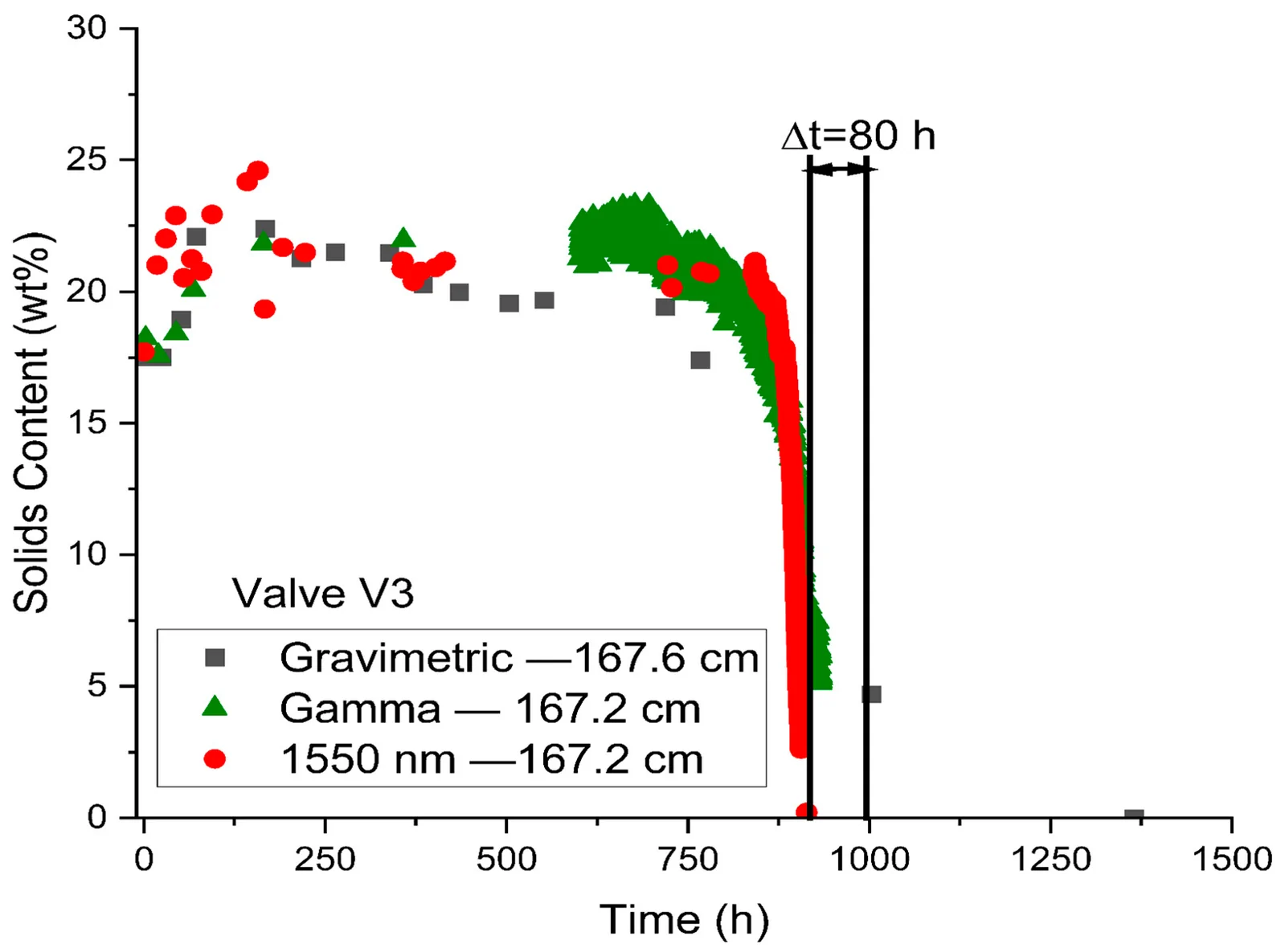
Temporal profiles of solids content measured at the V3 outlet valve by the 1550 nm optical (red circle) and γ-ray (green triangle) sensors of the ST-SCA and the gravimetric method on extracted samples (black square).

V3 is the second valve from the top. The solids content at V3 was measured by the 1550 nm optical sensor and the gamma-ray sensor of the ST-SCA and compared with the values obtained by the gravimetric method on extracted samples (Figure 11). The optical and gamma-ray sensors were located 170.2 cm from the bottom of the column. The FFT samples used for the gravimetric method were extracted at V3, which was located 167.6 cm from the column bottom. Good agreement can be observed between these three data sets. The optical and gamma-ray sensor data reached zero values earlier than the gravimetric method data. The time difference between the zero-value sensor data and the lowest non-zero gravimetric data was approximately Δt ≈ 80 h. The height difference between V3 and the optical sensor was Δh ≈ 2.6 cm. The mudline moved at a speed of v_ml ≈ 0.03 cm/h near the V3 location, as estimated from Figure 8; thus, Δh/v_ml ≈ 87 h. The time differences observed between the two measurements and that estimated based on the mudline speed are consistent with each other. The origin of the oscillations in the first 500 h of optical sensor data may be due to settling dynamics and requires further investigation. The non-zero values in the optical sensor data were attributed to ambient light. This issue was mitigated in a later modification of the optical sensor by adding a band-pass filter in front of the photodetector to block visible light transmission.

#### 3.2.4. V4 Valve Position Comparison

**Figure 12:**
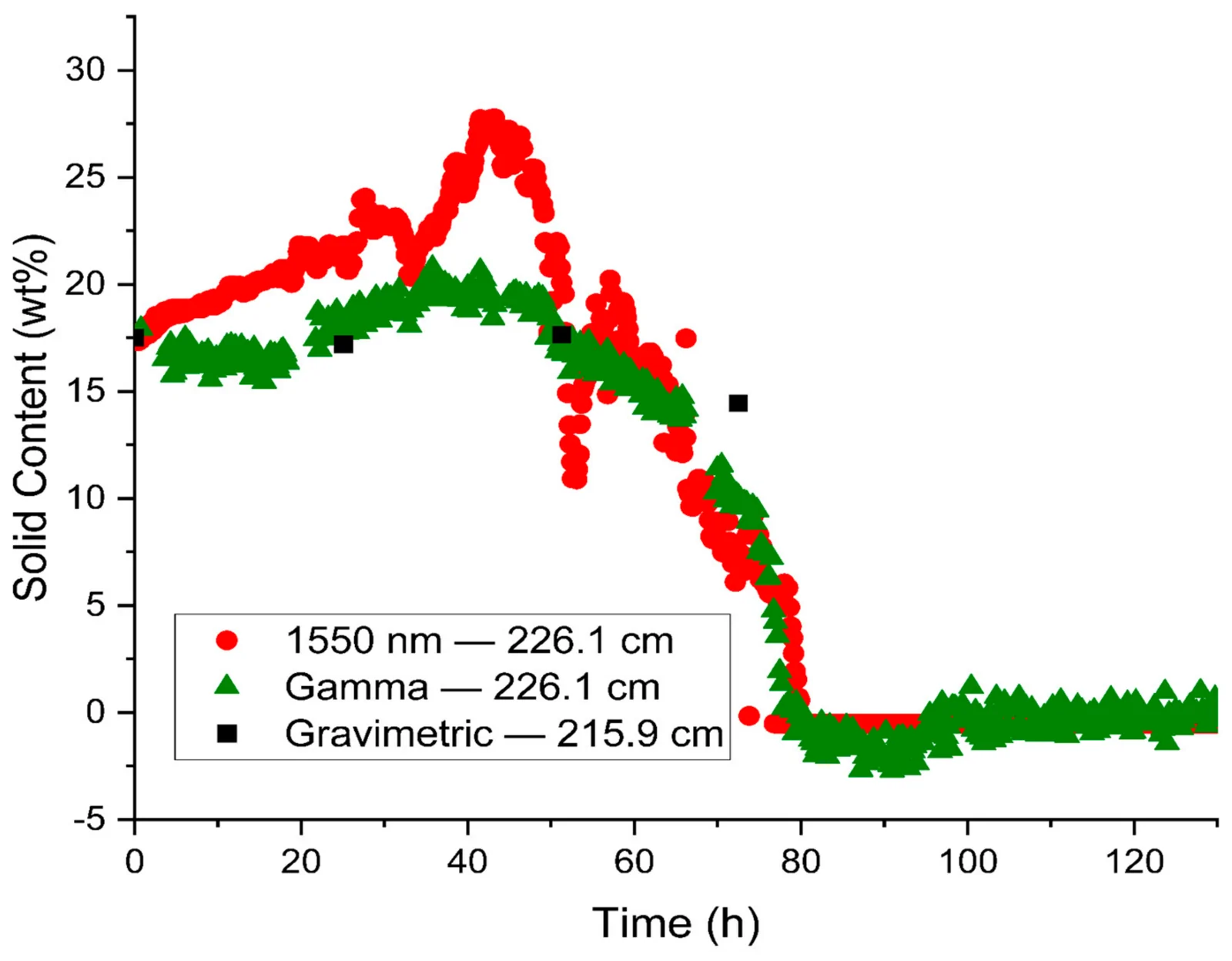
Temporal profiles of solids content measured at the V4 outlet valve position by the 1550 nm optical (red circle) and γ-ray (green triangle) sensors of the ST-SCA and the gravimetric method on extracted samples (black square).

As shown in Figure 12, temporal profiles of solids content near V4 were measured by the 1550 nm optical sensor and the γ-ray sensor of the ST-SCA and compared with those obtained by the gravimetric method on extracted samples. The optical and γ-ray sensors were located 226.1 cm from the bottom of the column. The FFT samples used for the gravimetric method were extracted using V4, which was located 215.9 cm from the bottom of the column. Good agreement can be observed between these three data sets. A step was evident in the optical and γ-ray data during the experimental period from 30 to 50 h; this was due to disturbance from taking the gravimetric sample, which caused mixing in the column near V4 with higher and lower weight percentages. The top layer exhibited more fluctuations, and the sample settled faster.

### 3.3. Temporal Profile of Solids Content with DS-SCA

The ST-SCA is useful for long-term monitoring of settling behavior and can scan continuously for extended periods of several months. The DS-SCA is designed to quickly scan locations in a settling pond in a few hours for rapid surveys.

#### 3.3.1. Valve V3 Comparison

**Figure 13:**
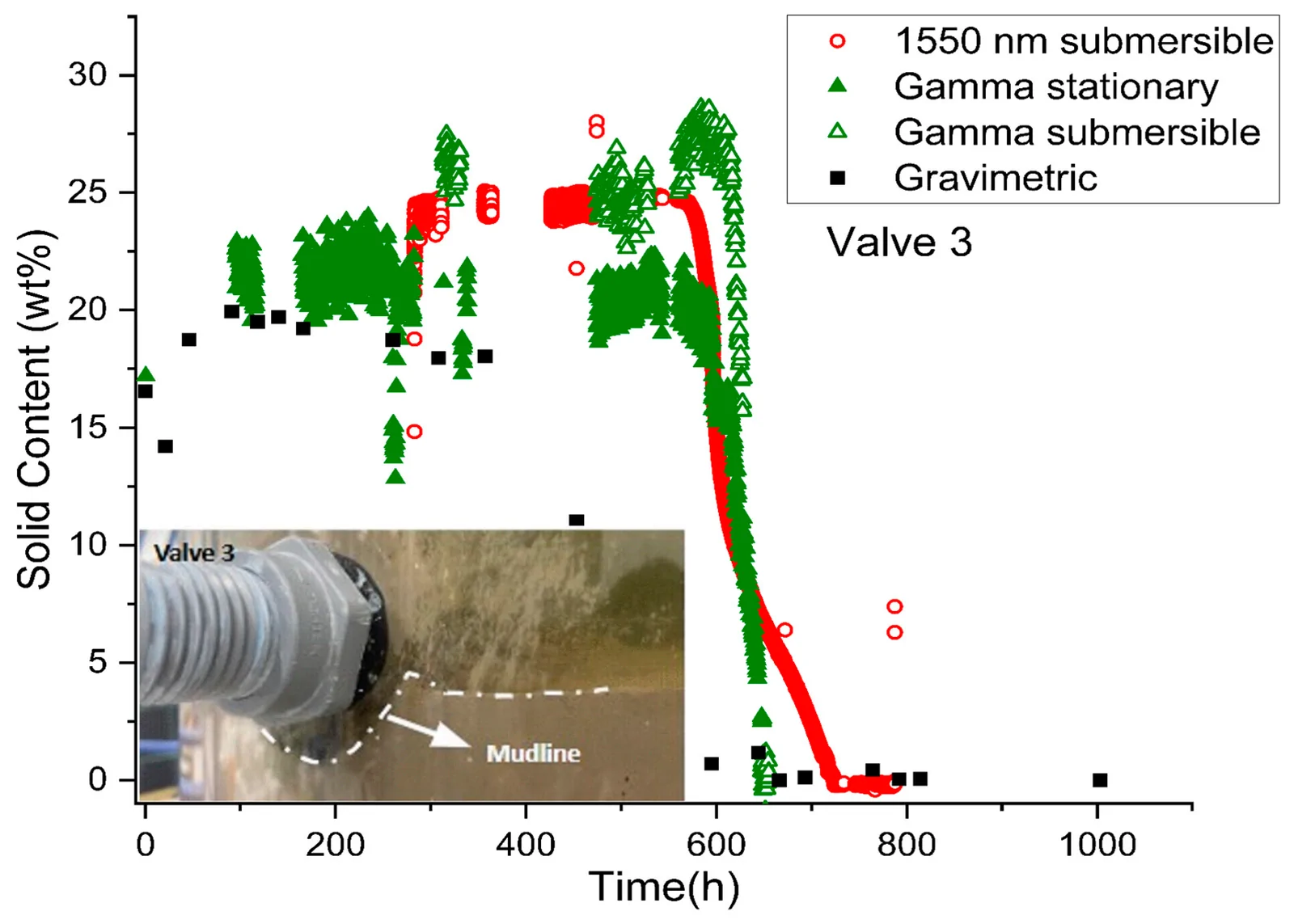
Temporal profiles of solids content measured at the V3 valve position by the 1550 nm optical and γ-ray sensors of the DS-SCA, γ-ray sensor of the ST-SCA, and the gravimetric method on extracted samples.

This section describes measurements made using the DS-SCA instrument. The temporal profiles of solids content at the V3 location were measured by the 1550 nm optical sensor and gamma-ray sensor of the DS-SCA, as well as the gamma-ray sensor of the ST-SCA (Figure 13), and compared with those obtained by the gravimetric method on extracted samples. The optical and gamma-ray sensors were positioned 168 cm from the column bottom. The FFT samples used for the gravimetric method were extracted at V3, located 167.6 cm from the column bottom. Good agreement can be observed between all data up to 250 h. Around 250 h, gravimetric sampling caused a dip in the sensor data. As shown in Figure 2, the DS-SCA was located on the left side of the column, while the ST-SCA gamma-ray sensor and the V3 sampling point were on the right side. The gravimetric sampling results in the ST-SCA gamma-ray sensor data and gravimetric method data show lower solids content values than the optical and gamma-ray sensor data from the DS-SCA. Additionally, the gravimetric method data exhibit early settling behavior due to the method’s poor depth resolution and the downward movement of more dilute tailings from above when sampling, as illustrated in the inset image in Figure 13. The DS-SCA is a heavier probe and can cause disturbances in the sample. The probe was moved slowly at around 1 cm per second to minimize disturbance. A minor transient change was seen in the stationary 1550 and 980 nm data when the DS-SCA passed through the monitored measurement levels. Transient changes of approximately 0.5 wt% were observed in the optical data as the probe was lowered to a nearby depth. However, the readings returned to the original value in approximately two hours.

#### 3.3.2. Valve V4 Comparison

**Figure 14:**
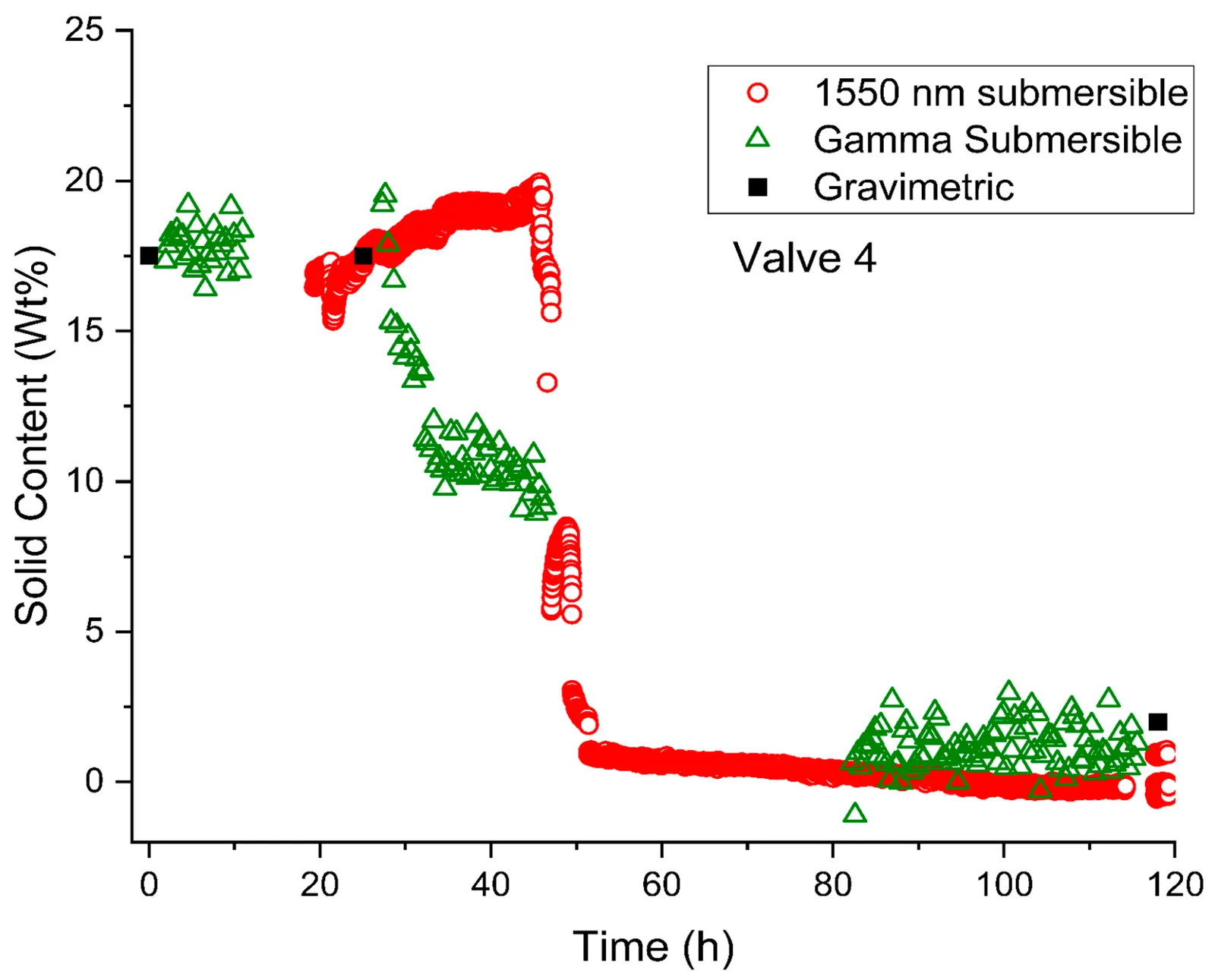
Temporal profiles of solids content measured at the V4 valve position by the 1550 nm optical and γ-ray sensors of the DS-SCA and the gravimetric method on extracted samples.

As shown in Figure 14, temporal profiles of solids content near V4 were measured using the 1550 nm optical sensor and the gamma-ray sensor of the DS-SCA and compared with those obtained by the gravimetric method on extracted samples. The optical and gamma-ray sensors were located 226.1 cm from the bottom of the column. The FFT samples used for the gravimetric method were extracted using V4, which was located 215.9 cm from the bottom of the column. Good agreement can be observed between these three data sets. A step was observed in the gamma-ray data during the period from 30 to 50 h. This step is absent in the optical sensor data due to apparent sample buildup on the window in front of the optical sensor, resulting in continuously high values during the 30- to 50-h period. When the sample fell off the window, the signal dropped rapidly, resulting in the sharp edge shown. The window of the optical sensor had a recessed opening that could occasionally trap some of the larger particles or aggregates.

### 3.4. Depth Profile of FFT Solids Content

**Figure 15:**
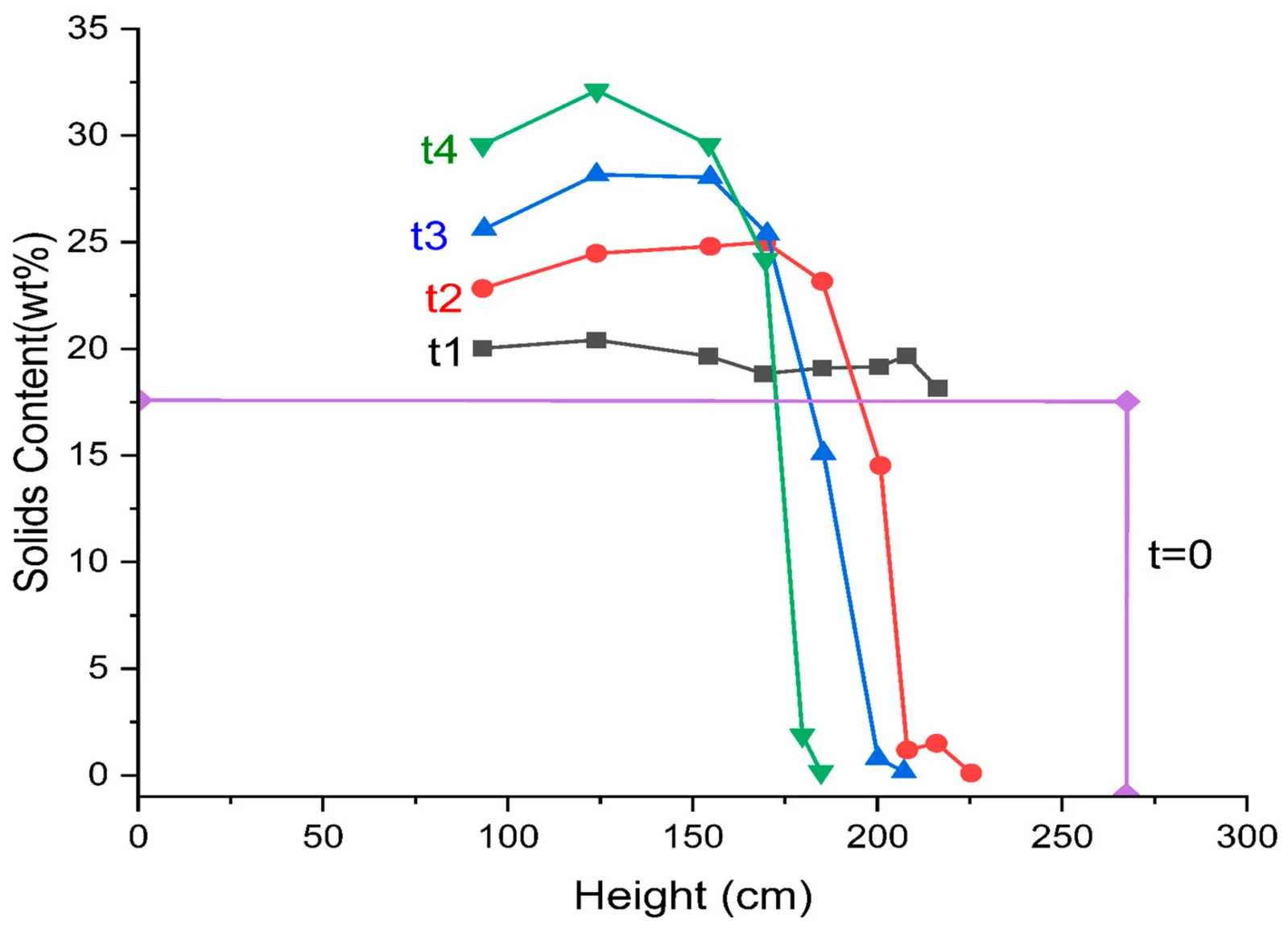
Solids content as a function of distance from the column bottom at various times was measured by the γ-ray sensor of the ST-SCA. The dark line is the initial solids content profile. The black, red, blue, and green profiles correspond to the following time periods: t1 (18.9–21 h), t2 (1163–166.5 h), t3 (357.3–358.9 h), and t4 (599.8–602.1 h), respectively.

The depth profiles of solids content at various times can be extracted from in situ sensor data. As shown in Figure 15, solids content as a function of depth at various times was measured by scanning the γ-ray sensor of the ST-SCA over a range of depths. As can be seen in Figure 15, the edge of the solids content profile moved toward the bottom of the column, and its maximum values increased from 17.5 wt% initially to over 30 wt% at around 600 h. These tests show that this sensor is capable of providing tailings pond survey companies with accurate depth profiles.

### 3.5. Downward Scanning with DS-SCA

**Figure 16:**
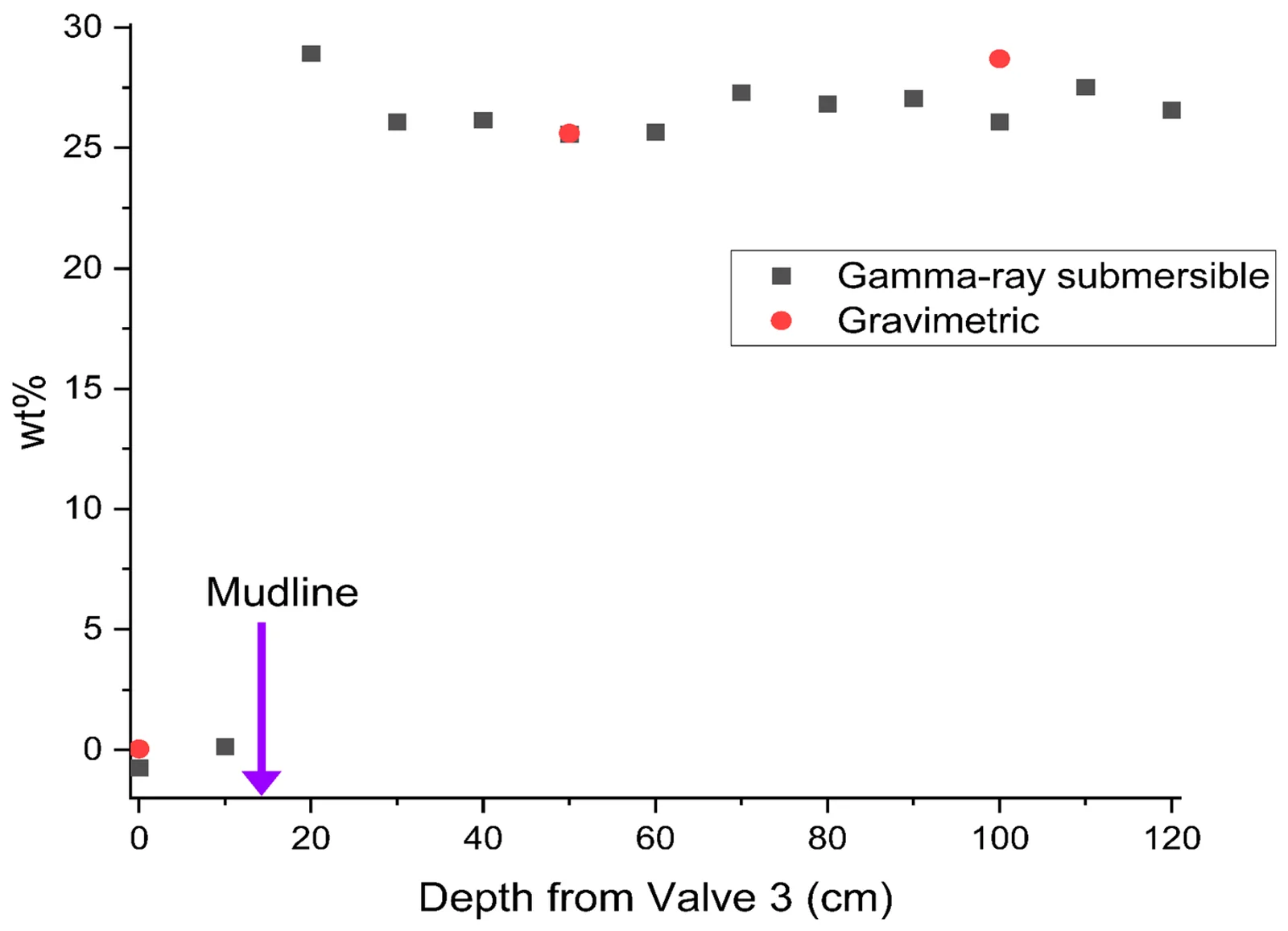
Solids content depth profile was measured by the γ-ray sensor (black squares) of the DS-SCA after approximately 800 h and compared with the gravimetric method data (red circles). The mudline location is indicated by the purple arrow.

The DS-SCA can be used to carry out depth scans. The DS-SCA was used to scan downward from the V3 location (167.6 cm from the column bottom), approximately 800 h after the start of the experiment. In the previous section, the submersible was kept stationary after lowering it down to a particular position. However, the submersible module was also tested by lowering it at intervals of 15 min to record depth-scan profiles. This experiment was performed to demonstrate the capability of the DS-SCA to scan different locations without fixing its position, demonstrating that it can be used anywhere at any time. As shown in Figure 16, the solids content depth profile was measured by the γ-ray sensor of the DS-SCA and compared with the gravimetric method data. Good agreement between the two sets of data can be observed. The mudline was estimated to be 152 cm above the column bottom (purple arrow in Figure 16). Good agreement between the solids content depth profile data and the mudline data can also be observed.

## 4. Discussion

In the current study, techniques that have been developed for monitoring solids content were integrated into two prototype measurement systems. The ST-SCA is a stationary column of detectors that can be used for continuous monitoring of the solids content profile in dynamic locations of tailings ponds where the solids profile may change continuously with ongoing operations. The DS-SCA is a portable submersible instrument that can be used for carrying out real-time scans of solids profiles at any location in a tailings pond. These systems have been tested in a 3 m settling column with real tailings samples obtained from an operating oil sands facility. It can be seen that the optical backscatter measurements at 1550 nm and gamma-ray measurements were capable of quantitatively measuring the solids content in the tailings samples with a high degree of accuracy. Accuracy was determined by comparison with the current standard of gravimetric measurements of tailings samples extracted at various time intervals and column heights as the solids settled over several months.

In previous studies [11], it was found that the 1550 nm wavelength is the optimum wavelength to use for scattered light measurements since it is relatively immune to bitumen fouling because the bitumen is transparent at this wavelength. This alleviates one of the most problematic issues for using optical diagnostics in oil sands tailings since bitumen fouling on windows will strongly absorb visible radiation. In general, increasing solids content will increase backscattering of light. At the same time, the 1550 nm wavelength is strongly absorbed by water. Therefore, at higher solids wt%, more light is scattered back to the detector before it can be significantly absorbed by the residual water. Consequently, the measured optical signal increases with wt% due to the reduction in the effective water path length from the scattering process. Overall, this leads to a relatively robust technique for measuring the solids content of fluid tailings.

In contrast, the 980 nm wavelength was strongly absorbed in residual bitumen in the sample and acted as a binary indicator of significant solids or the absence of solids. Previous measurements have shown that it is only sensitive over the range from 0 to 10 wt%. At higher solids content, the signal became saturated and no longer scaled with increasing solids content. Thus, the 980 nm wavelength acts as a sensitive indicator of the mudline layer as the solids content changes from <5% to >5% in the settling tailings.

For the optical measurements at the 1550 nm wavelength, calibration of the signal response versus solids content was carried out in an offline ring scattering setup of up to a value of 30 wt%. The resultant calibration curve was then normalized to the initial response measured by the in situ light scattering detectors at the 17.5 wt% point when the column was initially filled with the homogeneous sample. When comparing measured detector values with the gravimetric measurements over the measurement range of up to 25 wt%, the data were found to be in agreement within a standard deviation of 3 wt% for the optical system and within 2 wt% for the gamma-ray system. For accurate comparison, gravimetric results from Valve 3 are compared with Level 6, and Valve 4 results are compared with Level 2. The valves and sensors are at the same height for these two levels. Gravimetric results can also have some inaccuracies due to very limited depth resolution and disturbance from sampling and testing. Part of the difference can be accounted for by the level difference in height between the optical measurements and the drainage valves for the gravimetric measurements. Furthermore, drainage of the sample from valves can lead to some layer mixing. For the two lowest valve positions, the gravimetric measurements appeared consistently higher than the optical and gamma-ray measurements; this may be because the larger particles settled rapidly at first. Thus, the size distribution of particles may have shifted to larger values in these cases. The experiment was repeated three times. Every time, the sample settled faster than in the previous run. Heavy and thick particles settled at the bottom of the column and were difficult to remove. Fewer and larger particles may produce less optical scattering than a greater number of smaller particles at a similar wt%, leading to lower readings for the optical measurement. It could be that the atomic composition of larger particles is different from that of smaller particles, leading to different gamma-ray absorption and differences in the gamma-ray measurements at these depths. To estimate the measurement errors of the sensors, we compared the gravimetric measurements from valve V3 data with Level 6 sensor data, as the locations of both levels were the same. Hence, they were comparable. For the 1550 nm sensor, the differences between the gravimetric and sensor data for hours 79, 167, 222, 357, and 719 were 1.31, 3.04, 0.22, 0.32, and 1.6 wt%, respectively, leading to an rms standard deviation of 1.8%. For the gamma-ray sensor, the differences between gravimetric and sensor data for hours 18, 167, 357 and 719 were 0.54, 0.57, 0.48, and 1.52 wt%, respectively, leading to an rms standard deviation of 1.0%. Allowing for additional systematic errors, we estimated the rms error per measurement to be approximately 3 wt% for the 1550 nm measurements and 2 wt% for the gamma technique.

Window fouling by samples with higher solids content was observed a few times during the measurements. For instance, the laser submersible measurements from 30 to 50 h around level V4 showed evidence of window fouling, as shown in Figure 14. Generally, for the stationary and submersible measurements, the window would become clear after a period of tens of hours, and signals corresponding to the much more dilute samples were obtained.

For the gamma-ray measurements, a value for gamma-ray photon energy must be selected that provides measurable residual transmission through several centimeters of sample but also has enough attenuation to achieve reasonable absorption over a few centimeters of path length in samples with higher solids content. It was found that photon energies of approximately 30 keV were suitable for such measurements. A Ba-133 radioisotope source has X-ray emission lines at 30 and 80 keV. The transmission of 30 keV photons was used as an indicator of solids content. In this case, the higher average atomic number of the solids led to increased gamma-ray absorption. The 10.6-year half-life of Ba-133 will lead to a decline in signal strength over longer measurement periods. Because of the relatively short duration of the measurements reported here, no corrections were applied to the decay of the Ba-133 source strength data in this study. If a long-term experiment is carried out, for example, for more than a few months, then it is recommended to correct for the decay in source strength, which can be achieved based on the decay lifetime of Ba-133. The transmission of the 80 keV photons could also be used as a monitor of source strength, though this was not carried out in the present study. For the measurements, it is important to calibrate the detector for the actual geometry of the detector assembly since scattered gamma rays from the surrounding volume, including the detector housing, also contribute to the signal. In the present case, calibration at three solids content values was carried out, and a second-order calibration curve fit to these data was then used for the instrument response function. The response of each detector was then referenced to its corresponding value in air, accounting for variations in source strength and the exact collection geometry of each detector position. Alternatively, a previous study [31] found that variations in signal as a function of solids content can be obtained from GEANT4 Monte Carlo simulations of X-ray transmission and scattering using the known chemical composition of the sample. That work showed that the calculations agreed with physical measurements within a few wt% error. By optimizing the detector geometry and path length, it was found that a weak Ba-133 radioisotope source below the licensing regulatory limit was adequate to allow accurate measurements of solids content in a few minutes of sample acquisition. Although this can still be considered quick compared with physical sampling and gravimetric methods, it is significantly longer than the optical detectors, which have measurement times of tens of seconds. Therefore, it is proposed that the gamma-ray sources are used as cross-calibration measurements to ensure that values from the optical measurements are not affected by potential window fouling. For future development, a multi-detector array can be used to shorten the scanning time to a few seconds.

Overall, the gamma-ray measurements agree with the gravimetric measurements within a few wt%. One DS-SCA measurement exceeded the gravimetric measurement by several wt% in the period of 570 to 600 h at level V3. This may be attributed to the accumulation of some higher solids content samples in the measurement path of the gamma-ray sensor. Since the gamma-ray detector has one direct line of sight from the source to the detector element, it is sensitive to an anomalous portion of the tailings present in this path.

It is expected that the accuracy, speed, and reliability of the detector can be improved further. It is possible to increase the sampling rate of the optical detector since the photon signals are quite strong, and the current design was conservative in averaging readings over 10-s intervals. It is expected that optical sensor reading times can be decreased to a few seconds per measurement. For the gamma-ray sensor, it is proposed that multiple detectors are placed around one source, which would increase the net counting rate for a given source. Thus, a stronger radioactive source is not required, which would be undesirable from a safety perspective. Such improvements should be considered in future development of the detectors. Sample particle size can still have an effect on the 1550 nm scattered signal, and future studies can address corrections to the calibration curves for varying particle size distributions. However, by simultaneously measuring some data points with the 1550 nm sensor and gamma-ray sensor, the calibration curve can be corrected to first order to provide accurate readings since the gamma-ray sensor is not affected by particle size. Additionally, having both sensors allows for identification of bad readings, which may occur due to the significant fouling of one of the windows.

## 5. Conclusions

In the current study, the previously developed optical [11] and gamma-ray [31] techniques for measuring solids content in settling ponds were integrated into full operational systems that can be used for monitoring solids content in tailings ponds. A stationary solids content analyzer, ST-SCA, and a depth-scanning submersible analyzer, DS-SCA, were developed and validated under realistic operating conditions using a large pilot-scale 3 m column. It was shown that both techniques could accurately measure the solids content in tailings, with agreements within a few wt% compared with conventional gravimetric measurements on physical samples taken from the same column at various intervals. The scanning submersible analyzer enables rapid measurement of solids’ depth profiles without the need for time-consuming and costly sampling followed by subsequent laboratory analysis. The results indicate the sensor method has good capabilities in the characterization of the FFT solids content dynamics with good temporal resolution. The technology can be deployed in actual oil sands tailings ponds for real-time in situ measurements of solids content at different locations and depths. It is expected that this relatively low-cost technology can facilitate better monitoring and management of oil sands tailings, resulting in improved process efficiency and improved environmental performance.

## Data Availability

The original contributions presented in this study are included in the article. Further inquiries can be directed to the corresponding author.

## Abbreviations

The following abbreviations are used in this manuscript:

MFT	Mature Fine Tailing
FFT	Fine Fluid Tailing
ST-SCA	Stationary solids content analyzer
DS-SCA	Depth-scanning solids content analyzer
